# Supplementary material for: Assessing the completeness and accuracy of South African National Laboratory CD4 and viral load data: a cross-sectional study
Source: BMJ Open. 2018 Aug 23;8(8):e021506. doi: 10.1136/bmjopen-2018-021506 (PMC6112393; doi:10.1136/bmjopen-2018-021506)
Supplement: Supplementary data [file bmjopen-2018-021506supp001.pdf]

Supplementary Table 1. Sequence of Matching Criteria for Patient Identifiers

**Confident match**
Exact match on surname, first name, DOB*, gender
Exact match on surname, at least first word of first name, DOB, gender
Exact match on surname, first name and:<br>DOB (gender missing or unusable)<br>Gender (DOB missing or unusable)
Exact match on at least first word of surname, at least first word of first name, DOB, gender
Exact match on at least first word of surname, at least first word of first name and:<br>DOB (gender missing or unusable)<br>Gender (DOB missing or unusable)
**Likely match**
Surname and first name are reversed and:<br>Exact match on DOB and gender<br>Exact match on DOB (gender missing or unusable)<br>Exact match on gender (DOB missing or unusable)
First word of surname and first word of first name are reversed and:<br>Exact match on DOB and gender<br>Exact match on DOB (gender missing or unusable)<br>Exact match on gender (DOB missing or unusable)
**Likely match despite keying errors**
Exact match on surname, first name, DOB, gender different
Exact match on surname, first name, gender, DOB discrepant in one part (day, month, or year)
Exact match on surname, at least first word of first name, DOB, gender different
Exact match on surname, at least first word of first name, gender, DOB discrepant in one part (day, month, or year)
Exact match on first word of surname, at least first word of first name, DOB, gender different
Exact match on first word of surname, at least first word of first name, gender, DOB discrepant in one part (day, month, or year)
Surname and first name are reversed, exact match on DOB, gender different
Surname and first name are reversed, exact match on gender, DOB discrepant in one part (day, month, or year)
First word of surname and first word of first name are reversed, exact match on DOB, gender different
First word of surname and first word of first name are reversed, exact match on gender, DOB discrepant in one part (day, month, or year)
**Possible match (manual review required)**
Exact match on at least first word of surname, first word of first name does not match , exact match on DOB (if usable) and gender (if usable)
First word of surname does not match, exact match on at least first word of first name, DOB (if usable) and gender (if usable)
**Other match (manual review required)**

*DOB: date of birth.

Supplementary Table 2. Sequence of Matching Criteria for CD4 and Viral Load (VL) Tests

**Confident match**	
	Exact match on CD4 or VL value* and McCord test date consistent:<br>Exact match on test date<br>Month and day of test date reversed<br>Test date within 7 days<br>Test date discrepant in one part (day, month, or year)<br>Exact match on registration date<br>Month and day of registration date reversed<br>Registration date within 7 days<br>Registration date discrepant in one part (day, month, or year)
**Possible match**	
	Different CD4 or VL value, exact match on test date
	Different CD4 or VL value, exact match on registration date
**Unlikely match**	
	Exact match on CD4 or VL value, different test date
	Exact match on CD4 or VL value, different registration date
**No match**	
	Different CD4 or VL value, different test and registration dates
	No CD4 or VL value in NHLS

* VL values are considered matched on the value in any of the following situations:
1. Both McCord and NHLS records had matching viral load values
2. McCord record had a value of <150 copies/ml and NHLS record value was marked “<150”
3. McCord record had a value of <40 copies/ml and NHLS record value was marked “<40”
4. McCord record had a value of <20 copies/ml and NHLS record value was marked “<20”
5. McCord record value was marked “undetectable” and the NHLS record value was marked “<150”, “<40”, “<20”, or “lower than detectable limit”

SUPPLENTARY FIGURE LEGEND

Supplementary Figure 1A. Process of Determining Cohorts for Crossmatching Analysis

We started with a patient list of 4257 McCord Hospital study IDs. Prior to matching with NHLS data, we removed duplicated study IDs (n=12), patients <18 years old on June 30, 2012 (n=337), and patients who had neither a CD4 count nor VL record from McCord Hospital (n=2), leaving a cohort of 3906 patients for patient identifier matching ("Filter 1"). For the CD4 matching analysis, we then removed a patient who did not have a CD4 count record from McCord Hospital (n=1), leaving a cohort of 3905 patients for CD4 matching ("Filter 2"). For the VL matching analysis, we removed 297 patients who did not have a VL record from McCord Hospital, leaving a cohort of 3609 for VL matching ("Filter 3").

Abbreviations: **NHLS:** National Health Laboratory Services; **VL:** viral load.

Supplementary Figure 1B. Process of Receiving NHLS Data for Crossmatching Analysis

We sent 4257 McCord Hospital study IDs to the NHLS. Study IDs were sent with associated patient identifiers (first name, surname, gender, date of birth) and last recorded CD4/VL from McCord Hospital. The NHLS then returned 3774 study IDs; the returned dataset contained 16 340 CD4 records and 18 677 VL records from 3774 patients. We then compared these 3774 study IDs to each of our filtered cohorts (Supplemental Figure 1A). Of our 3906 cohort for patient identifier matching, 3498 had one or more records returned by NHLS. Of our 3905 cohort for CD4 matching, 3451 had one or more CD4 records in NHLS. Of our 3609 cohort for VL matching, 3365 had one or more VL records in NHLS.

Abbreviations: **NHLS:** National Health Laboratory Services; **VL:** viral load.

Supplementary Figure 1A.

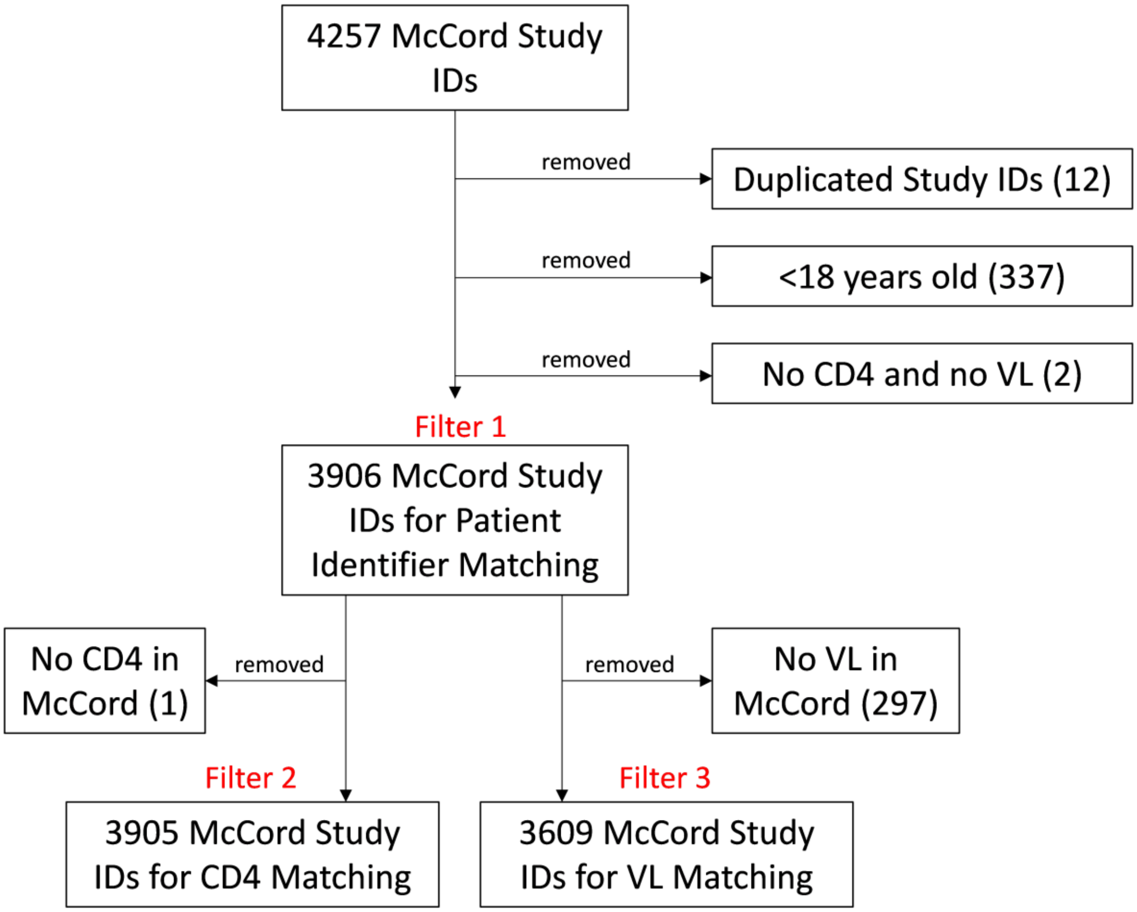

Supplementary Figure 1B.

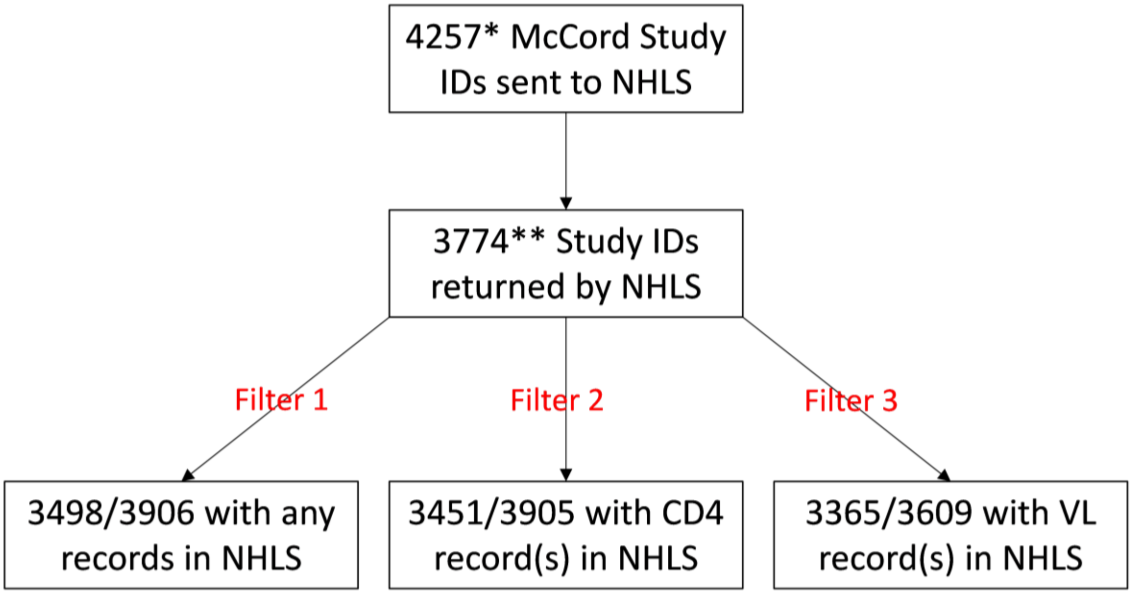

*Study IDs sent with associated patient identifiers and last recorded CD4/VL from McCord Hospital.
**Dataset contained 16 340 CD4 records and 18 677 VL records from 3774 patients.
