# Supplementary material for: Assessing the completeness and accuracy of South African National Laboratory CD4 and viral load data: a cross-sectional study
Source: BMJ Open. 2018 Aug 23;8(8):e021506. doi: 10.1136/bmjopen-2018-021506 (PMC6112393; doi:10.1136/bmjopen-2018-021506)
Supplement: Author's manuscript [file bmjopen-2018-021506.draft_revisions.pdf]

BMJ Open

BMJ Open is committed to open peer review. As part of this commitment we make the peer review history of every article we publish publicly available.

When an article is published we post the peer reviewers' comments and the authors' responses online. We also post the versions of the paper that were used during peer review. These are the versions that the peer review comments apply to.

The versions of the paper that follow are the versions that were submitted during the peer review process. They are not the versions of record or the final published versions. They should not be cited or distributed as the published version of this manuscript.

BMJ Open is an open access journal and the full, final, typeset and author-corrected version of record of the manuscript is available on our site with no access controls, subscription charges or pay-per-view fees (http://bmjopen.bmj.com).

If you have any questions on BMJ Open's open peer review process please email info.bmjopen@bmj.com

BMJ Open

Assessing the Completeness and Accuracy of South African National Laboratory CD4 and Viral Load Data

Journal:	*BMJ Open*
Manuscript ID	bmjopen-2018-021506
Article Type:	Research
Date Submitted by the Author:	03-Jan-2018

Complete List of Authors:	Bassett, Ingrid; Massachusetts General Hospital, Division of Infectious Disease; Harvard Medical School<br>Huang, Mingshu; Massachusetts General Hospital, Division of General Internal Medicine; Massachusetts General Hospital, Biostatistics Center<br>Cloete, Christie; McCord Hospital<br>Candy, Sue; National Health Laboratory Service<br>Giddy, Janet; McCord Hospital<br>Frank, Simone; Massachusetts General Hospital, Division of General Internal Medicine; Massachusetts General Hospital, Medical Practice Evaluation Center<br>Parker, Robert; Massachusetts General Hospital, Biostatistics Center; Harvard Medical School

Keywords:	South Africa, National Health Laboratory Services (NHLS), national laboratory systems, HIV record matching, data crossmatching method, CD4 and viral load

SCHOLARONE™ Manuscripts

only

Assessing the Completeness and Accuracy of South African National Laboratory CD4 and Viral Load Data

Running head: Evaluation of national laboratory data

Ingrid V. Bassett, MD, MPH,[1,2,3,4,5] Mingshu Huang, PhD,[2,3,4,6] Christie Cloete, MBChB,[7] Sue Candy, BSc,[8] Janet Giddy, MBChB, MFamMed,[7] Simone C. Frank, BA,[2,3] Robert A. Parker, ScD[2,3,4,5,6]

[1]Division of Infectious Disease, Massachusetts General Hospital, Boston, Massachusetts, United States of America

[2]Division of General Internal Medicine, Massachusetts General Hospital, Boston, Massachusetts, United States of America

[3]Medical Practice Evaluation Center, Department of Medicine, Massachusetts General Hospital, Boston, Massachusetts, United States of America

[4]Harvard University Center for AIDS Research (CFAR), Boston, Massachusetts, United States of America

[5]Harvard Medical School, Boston, Massachusetts, United States of America

[6]Biostatistics Center, Massachusetts General Hospital, Boston, Massachusetts, United States of America

[7]McCord Hospital, Durban, South Africa

[8]Corporate Data Warehouse, Department of Information Technology, National Health Laboratory Services, Johannesburg, South Africa*

For peer review only - http://bmjopen.bmj.com/site/about/guidelines.xhtml

*Current position: National Institute of Communicable Diseases, National Health Laboratory Service, Johannesburg, South Africa

Correspondence:

Ingrid V. Bassett, MD, MPH

Massachusetts General Hospital

50 Staniford Street, 9th Floor

Boston, MA 02114

Tel +1 617 726 0637

IVB: ibassett@partners.org

MH: jenniferhuangmsh@gmail.com

CC: christie_cloete@hotmail.com

SC: sue.candy@nhls.ac.za

JG: janet.giddy@gmail.com

SCF: SCFRANK@mgh.harvard.edu

RAP: RPARKER4@PARTNERS.ORG

Word count: 3,013 (word limit 4,000)

These data were presented in part at the 21st International AIDS Conference (AIDS 2016) July 18-22, 2016 in Durban, South Africa.

Keywords: South Africa, National Health Laboratory Services (NHLS), national laboratory systems, HIV record matching, data crossmatching method, CD4 and viral load

For peer review only - http://bmjopen.bmj.com/site/about/guidelines.xhtml

ABSTRACT (word count: 271; word limit: 300)

Objective: To assess the accuracy of the South African National Health Laboratory Services (NHLS) centralized data warehouse (CDW) using a novel data crossmatching method.

Methods: Adults (≥18y) on antiretroviral therapy who visited a hospital-based HIV clinic in Durban from March-June 2012 were included. We matched patient identifiers, CD4 and viral load (VL) records from the HIV clinic's electronic record with the NHLS CDW according to a set of matching criteria for patient identifiers, test values and test dates. We calculated the matching rates for patient identifiers, CD4 and VL records, and an overall matching rate.

Results: NHLS returned records for 3498 (89.6%) of the 3906 individuals requested. Using our computer algorithm, we confidently matched 3278 patients (83.9% of the total request). Considering less than confident matches as well, and then manually reviewing questionable matches using only patient identifiers, only 9 (0.3% of records returned by NHLS) of the suggested matches were judged incorrect.

Conclusions: We developed a data crossmatching method to evaluate national laboratory data and were able to match almost nine of ten patients with data we expected to find in the NHLS CDW. We found few questionable matches, suggesting that manual review of records returned was not essential. As the number of patients initiating ART in South Africa grows, maintaining a comprehensive and accurate national data repository is of critical importance, since it may serve as an invaluable tool to evaluate the effectiveness of the country's HIV care system. This study

For peer review only

For peer review only - http://bmjopen.bmj.com/site/about/guidelines.xhtml

helps validate the use of NHLS CDW data in future research on South Africa’s HIV care system and may inform analyses in similar settings with national laboratory systems.

For peer review only - http://bmjopen.bmj.com/site/about/guidelines.xhtml

STRENGTHS AND LIMITATIONS OF THIS STUDY

- This is the first analysis to propose a novel method for examining the completeness and accuracy of records related to HIV care from a national data source.
- We developed a comprehensive and self-contained algorithm that may inform future analyses focusing on linkage to and retention in HIV care, and this methodology may also apply to data matching analyses in similar settings, as many sub-Saharan African countries have some sort of national laboratory system.
- NHLS requirements for submitting identifiers with laboratory requisitions during the study period were not strict enough to allow uniformly perfect matching; thus, we had to create extensive matching categories to cover the range of match types and quality.
- While we considered our patient identifier, CD4 and VL test record matching criteria detailed and comprehensive, a different team might develop an alternative set of rules and designations, and classify specific results differently.
- We had a large range of patient identifier matching criteria for what we considered an adequate match; while these criteria were discussed at length, they ultimately were subjective decisions.

For peer review only

For peer review only - http://bmjopen.bmj.com/site/about/guidelines.xhtml

INTRODUCTION

South Africa has the largest HIV treatment program in the world, with > 3.1 million people on antiretroviral therapy (ART) [1]. The government has expanded its national program in recent years in a transition to "country ownership" from the previous non-governmental organizations and private clinics [2-5]. As HIV care transitions to the public sector and the number of patients initiating ART grows, maintaining comprehensive and accurate patient data is of critical importance. Reliable and valid national data becomes increasingly useful for evaluating linkage to and retention in HIV care, for monitoring patients longitudinally across clinic sites, and for assessing the quality of care at the national level.

Patients undergoing HIV treatment at public and semi-private health centers in South Africa have routine blood samples sent to a National Health Laboratory Service (NHLS) laboratory for testing; these data are then stored at a central repository in the NHLS Corporate Data Warehouse (CDW). NHLS data have previously been used to evaluate the effectiveness of certain government-funded HIV programs [6-8], identify patterns of the TB epidemic [9, 10], and determine cancer incidence rates among HIV-infected individuals [11]. CD4 count and viral load (VL) records serve as indicators of being in HIV care, as these are monitored regularly while patients are receiving ART. However, NHLS CDW data have not been assessed to determine utility specifically for identifying and tracking patients in HIV care. While previous studies have compared mortality records between South African civil registration and clinics to evaluate the completeness of national mortality data [12, 13], no such comparison has been performed between CD4 and VL records for patients in HIV care.

For peer review only - http://bmjopen.bmj.com/site/about/guidelines.xhtml

We assessed the completeness and accuracy of the NHLS CDW for tracking patients using a cohort of patients who visited McCord Hospital's HIV clinic during a three-month period just prior to clinic closure due to loss of funding. We present here a method developed to match patients based on McCord Hospital patients' identifiers, CD4 records and VL values prior to transfer to data provided to us by the NHLS.

For peer review only - http://bmjopen.bmj.com/site/about/guidelines.xhtml

METHODS

Study Site

McCord Hospital was a semi-private, general hospital in KwaZulu-Natal serving a predominantly urban population from the greater Durban area. The Sinikithemba HIV clinic at McCord, which became a PEPFAR-funded site in 2004, was an integral part of the South African ART scale-up and initiated over 10 000 patients on ART [14]. Sinikithemba served a predominantly African, Zulu-speaking population. The clinic had a monitoring and evaluation team and an electronic medical record. Due to loss of PEPFAR funding, the clinic closed in 2012.

All patients who returned to the clinic for clinical appointments, laboratory tests, or pharmacy refills March 12-June 30, 2012 were referred for transfer to clinics in the Durban area. Data collected at the time of transfer included name, gender, date of birth, most recent pre-transfer CD4 count and VL values and dates. We have previously reported on the Sinikithemba transfer process evaluating linkage to initial transfer clinic visit and patient attitudes about their transfer experience using telephone surveys and clinic visits [14, 15].

Study Population

We studied adults ≥18 years on ART who visited the HIV clinic during the transfer period. Routinely collected programmatic data were used. Participants provided verbal consent for study participation. The study protocol was approved by the McCord Hospital Research Ethics

For peer review only - http://bmjopen.bmj.com/site/about/guidelines.xhtml

Committee (Durban, South Africa) and the Partners Human Research Committee (2012-P-001122/1, Boston, MA).

National Health Laboratory Service (NHLS)

The National Health Laboratory Service (NHLS) was established in 2001 and supports national and provincial health departments in South Africa. It is the largest diagnostic pathology service in the country, providing laboratory and related services to over 80% of the population through a national network of laboratories [6]. The NHLS performs all public sector CD4 and VL monitoring and maintains a CDW that serves as a national repository for laboratory data from the public sector. Healthcare workers at public health facilities complete laboratory requisition forms which accompany each sample submitted to the CDW. All data, including patient identifiers, name of facility, date of sample, and tests requested, are sent to the CDW and are captured electronically by the NHLS information system in real time. The CDW has developed an algorithm which utilizes both rules-based and probabilistic matching based on demographic attributes using fuzzy logic [16, 17]. This is applied to all test data at time of entry and results in a master patient index within the CDW.

Data Collection and Processing

We sent a list of all 4257 McCord Hospital transfer patients with corresponding identifiers (a patient ID internal to our population, used to identify "matches" between the NHLS and McCord datasets; first name; surname; sex; date of birth) to the NHLS for matching of laboratory records. The NHLS extracted data in October 2014. To assist with the matching process, we also sent last known CD4 and VL values and dates recorded in the electronic medical record at McCord

For peer review only - http://bmjopen.bmj.com/site/about/guidelines.xhtml

For peer review only

Hospital. We received two datasets (CD4 count and VL) containing potential matches from the NHLS. These datasets had 16 340 and 18 677 records from 3774 patients and included our internal patient ID, which reflected the patient that the NHLS believed that the records matched. We performed three separate matching analyses using patient identifiers (first name, surname, date of birth, gender), CD4 counts and test dates, and VL values and test dates. In each analysis, we assessed the quality of the match within our internal patient ID; thus, we assessed how well the data provided by the NHLS using their probabilistic matching technique represented a true match. From the original 4257 patient list, duplicated patient IDs (n = 12) and patients <18 years on June 30, 2012 (n = 337) were removed prior to matching. Two patients who had neither a CD4 count nor VL record from McCord Hospital were also removed. This left a cohort of 3906 patients to match based on patient identifiers. For the CD4 matching analysis, we removed 1 patient who did not have CD4 data in the McCord database, for a cohort of 3905 patients. We removed 297 patients who did not have VL data in the McCord database, resulting in a cohort of 3609 patients for the VL record matching analysis.

Matching of Records between NHLS and McCord Datasets

We performed our matching analysis in three stages; first, we cross-checked patient identifiers between the McCord and NHLS datasets to determine the distribution of optimal identifier matching, using all records for a particular individual prior to clinic closure. Next, we assessed the reported CD4 and VL records separately, independent of patient identifiers. Lastly, we considered the best test record match from a particular internal patient ID number in conjunction with the patient identifier match for that specific record to determine the overall distribution of matching based on both test records and patient identifiers. In this final matching analysis, the

For peer review only - http://bmjopen.bmj.com/site/about/guidelines.xhtml

patient identifier match was determined for the better match on either CD4 or VL. If the test match quality was the same, we used the better patient match of the two test records.

Matching Using Patient Identifiers

Within each internal patient ID, we used surname, first name, DOB and gender to assess the quality of the match between the NHLS CDW and the McCord data record. Based on a detailed set of matching criteria (Supplementary Table 1), we classified patient IDs into five general matching categories: *confident, likely, likely despite keying errors, possible*, and *other*. If corresponding patient identifiers fell into the latter two categories, they were reviewed manually; otherwise they were considered an adequate match and not reviewed. The manual review processes consisted of an independent review by two authors (IVB; SCF), with a third "tiebreaker" review by another author (RAP) for any discordant matching designations.

Matching Based on Test Results

We had a cohort of 3905 patients for the CD4 record matching analysis and 3609 patients for the VL matching analysis. If the CD4 count in the McCord record and a corresponding NHLS CDW record were an exact match, we compared the McCord test data to the two dates provided by the NHLS (test date and record date) for consistency (Supplementary Table 2). When the dates were consistent (exact match; month and day reversed; dates differed by less than 7 days; dates differed by one of year, month, or day), we considered the records a *confident* match. If the CD4 counts from corresponding McCord and NHLS CDW records differed, but there was an exact match on dates, we considered the records a *possible* match. If the dates did not match, we considered the records an *unlikely* match, even if the CD4 values matched. Records containing

For peer review only - http://bmjopen.bmj.com/site/about/guidelines.xhtml

both discrepant CD4 values and mismatching dates were not considered matched. Following these same criteria, we categorized corresponding NHLS CDW and McCord VL records as *confident*, *possible*, or *unlikely* matches. Because VL is often reported as undetectable, we had to use a somewhat looser criterion for considering the VL result an exact match (Supplementary Table 2).

Matching Based on Patient Identifier, Conditional on Matching based on a Test Result

After matching CD4 and VL values and dates, we assessed the accuracy of the patient identifier information based on the specific record used for the test matching. When there were equally good matches for both the CD4 and VL test, we used the better of the two patient matches for this classification.

For peer review only

For peer review only - http://bmjopen.bmj.com/site/about/guidelines.xhtml

RESULTS

Cohort Characteristics

Of 3906 participants included in the analysis, 41% of the cohort was male and the median age was 39 (interquartile range [IQR] 34 to 46). The majority of patients had CD4 counts above 200/µl at transfer (> 500/µl 29%, 200-500/µl 55%, < 200/µl 15%), and 84% of patients were virologically suppressed.

Best Patient Identifier Matching

Of 3906 patients, 3498 had one or more records returned by the NHLS. There were a median of 6 records (interquartile range: 5-7) per patient combining both CD4 and VL data; the maximum was 37 records for one individual. 3278 (93.7%) of these 3498 patients were considered *confident* matches. The distribution of patient identifier match categories is included in Table 1. Despite considering multiple potential matching criteria, only 45 additional matches (1.2%; *likely* and *possible* matches) were identified using automated procedures. Most of the additional matches (166; 4.7%) required manual review. Only 9 individuals (5.1%) of 175 who required manual review for the best match were not considered a match. Thus, only 0.3% of 3498 with any records were not considered matches. However, an additional 408 (10.4%) of the patients from McCord's HIV clinic did not have records in the NHLS CDW. Thus, overall we were able to match 89.3% of the patients in the McCord record with patients in the NHLS database, and virtually all of the records (99.7%) returned from NHLS were matches to the McCord patients.

Matching Based on CD4 Test Result and Date

For peer review only - http://bmjopen.bmj.com/site/about/guidelines.xhtml

After removing the 1 patient who did not have a CD4 test result in the McCord dataset, there were 3451 patients who had ≥1 CD4 records found in the NHLS CDW. 3270 (94.8%) of these 3451 patients had CD4 records that were considered a *confident* match. 57 (1.7%) records were considered *possible* matches and 36 (1.0%) were considered *unlikely* matches. There were 88 records (2.5%) which did not match on test value and did not match on test date. The distribution of CD4 record matching is shown in Table 2.

Matching Based on Viral Load Test Result and Date

After removing 297 patients who did not have VL results in the McCord dataset, there were 244 (6.8%) patients who did not have any VLs found in the NHLS CDW. Among the returned records for the remaining 3365 patients, there were 3306 (98.2%) VL records that were considered a *confident* match, 11 (0.3%) that were considered *possible* matches and 1 (0.03%) that was considered an *unlikely* match. There were 47 records (1.4%) which did not match on test value and did not match on test date. The distribution of VL record matching is shown in Table 3.

Quality of Patient Identifier Match for Best Test Record Match

After determining the best match for each test for a specific patient ID, we assessed how well the patient identifiers matched on the specific test record. Among the 3469 patients with a confident match on CD4 or VL, 3187 patients (91.9%) were also considered a *confident* match on the patient identifiers as well, and overall only 10 (0.3%) were not considered matched on the patient identifiers after manual review. Even the *possible* matches were found to be valid most of the time (185/189, 97.9%) after manual review, but only 23/29 (79.3%) of the *other* records were

For peer review only - http://bmjopen.bmj.com/site/about/guidelines.xhtml

valid matches. Most of the additional 272 matches were based on manual review (208/272, 76.5%). Overall, we manually reviewed 218 records, 10 of which were considered not matched (4.6%). The distribution of patient identifier matches by best test matches is shown in Table 4.

For peer review only

For peer review only - http://bmjopen.bmj.com/site/about/guidelines.xhtml

DISCUSSION

We assessed the completeness and accuracy of the NHLS CDW by matching patient identifiers and CD4 and VL test results from a McCord Hospital dataset to data returned by NHLS for these individuals. NHLS returned records for 89.6% of the individuals requested. Importantly, we found a very low false matching rate in the NHLS data, as only 0.3% of the patients identified by NHLS were not the patients from our initial request. Using only personal identifiers, we confidently matched 3278 of 3906 (83.9%) patients. Ignoring identifiers, we confidently matched 83.7% (3270 of 3905) of patients based on CD4 value and test date, and 91.6% (3306 of 3609) of patients with a VL result from McCord Hospital. Of all patients who had a confident match on either a CD4 or VL test, 91.9% (3187 of 3469) of those specific records were also a confident match using patient identifiers.

Comparing patient identifiers between McCord and NHLS datasets, a vast majority of patients were identified as *confident* matches. Confident matches made up 94% of the matched cohort, while all other matching categories combined (*likely*, *likely despite keying errors*, *possible*, and *other*) comprised only 6%, suggesting that the overall quality of matched records was high. While it was valuable to examine all potential match types and ranges of match quality, the extensive matching categories may not be necessary as the NHLS records returned were virtually always (99.7%) the patient for whom we requested data. When analyzing CD4 and VL test results separately, there was a slightly higher confident matching rate (98.2%) for VL results than for CD4 records (94.8%) among those with any results returned by NHLS. Patients considered a *confident* match in the CD4 analysis had to have an exact CD4 value match, while

For peer review only - http://bmjopen.bmj.com/site/about/guidelines.xhtml

patients in the VL analysis had to exhibit a match in VL status to be considered a *confident* match. Because VL results for most individuals are grouped into a suppressed category, the CD4 analysis may provide a more accurate matching process due to the more precise measure of CD4 value.

There are several limitations to our record matching method. NHLS requirements for submitting identifiers with laboratory requisitions during the study period were not strict enough to allow uniformly perfect matching; thus, we had to create extensive matching categories to cover the range of match types and quality. While we considered our patient identifier, CD4 and VL test record matching criteria detailed and comprehensive, a different team might develop an alternative set of rules and designations, and classify specific results differently. Additionally, we had a large range of patient identifier matching criteria for what we considered an adequate match; while these criteria were discussed at length, they ultimately were subjective decisions. While we were able to categorize a large proportion of records by our matching algorithm, there were additional records that required manual review. Although some manual matches could potentially have been more accurately resolved by consulting an outside source, we sought to keep the record matching algorithm self-contained to increase the likelihood that this method could be used by others. Finally, providing laboratory data to NHLS for the matching process might have improved the ability of the NHLS CDW to identify and match our specific patients, so our results might overestimate the ability to match records based solely on patient identifiers.

Despite the drawbacks of this methodology, this study has several important strengths. This is the first analysis to propose a novel method for examining the completeness and accuracy of

For peer review only - http://bmjopen.bmj.com/site/about/guidelines.xhtml

records related to HIV care from a national data source. We developed a comprehensive and self-contained algorithm that may inform future analyses focusing on linkage to and retention in HIV care. This methodology may also apply to data matching analyses in similar settings, as many sub-Saharan African countries have some sort of national laboratory system [18]. Due to the closing of the HIV clinic at McCord Hospital and the rapid transfer of a large cohort of patients, we had a considerable number of comprehensive and up-to-date records with which to assess the quality of NHLS CDW data.

For peer review only

CONCLUSION

As South Africa's HIV treatment program transitions to the public sector and the number of patients initiating ART grows, maintaining a comprehensive and accurate national data repository is of critical importance, as it may serve as an invaluable tool to evaluate the effectiveness of the country's HIV care system. Through the method that we created to evaluate national laboratory data, we have demonstrated that the NHLS CDW is both comprehensive and accurate. The NHLS CDW is centralized, broad, and supports a wide coverage of public clinics across the country; it therefore may serve as an appropriate and effective resource for tracking patients within the public HIV care system. Our ability to confirm the NHLS CDW as a reliable data source can help transcend the limitations of collecting and analyzing data within individual clinics, which presents challenges such as differences in record-keeping methods and marked variability in how patients are identified. This analysis not only validates the use of NHLS CDW data in future studies evaluating South Africa's HIV care system, but may also inform data matching projects in similar settings with national laboratory systems.

For peer review only - http://bmjopen.bmj.com/site/about/guidelines.xhtml

ETHICAL APPROVAL

Participants provided verbal consent for study participation. The study protocol was approved by the McCord Hospital Research Ethics Committee (Durban, South Africa) and the Partners Human Research Committee (2012-P-001122/1, Boston, MA).

For peer review only

For peer review only - http://bmjopen.bmj.com/site/about/guidelines.xhtml

FUNDING

This work was supported by the National Institutes of Health [R01 MH108427 and R01 MH090326-03S1] and the Harvard University Center for AIDS Research [P30 AI060354], which is supported by the following NIH Co-Funding and Participating Institutes and Centers: NIAID, NCI, NICHD, NIDCR, NHLBI, NIDA, NIMH, NIA, NIDDK, NIGMS, NIMHD, FIC, and OAR. The content is solely the responsibility of the authors and does not necessarily represent the official views of the National Institutes of Health.

For peer review only

For peer review only - http://bmjopen.bmj.com/site/about/guidelines.xhtml

COMPETING INTERESTS

The authors have no competing interests to declare.

For peer review only

For peer review only - http://bmjopen.bmj.com/site/about/guidelines.xhtml

AUTHORS' CONTRIBUTIONS

All authors have contributed significantly to this work and have reviewed and approved of this manuscript. IVB, Principal Investigator of this project, led the design and execution of this study as well as all stages of manuscript writing and preparation. MH and RAP led all data analysis efforts. MH initially helped to develop the novel data crossmatching method presented in the manuscript, while RAP also contributed substantially to and oversaw all method development. CC and JG both played significant roles in initial data collection and the procurement of records from McCord Hospital. SC also played a significant role in the procurement of CD4 and viral load records from the National Health Laboratory Services, which were used in the data crossmatch. SCF, the Research Assistant, contributed significantly to manuscript writing, editing, and review.

For peer review only

For peer review only - http://bmjopen.bmj.com/site/about/guidelines.xhtml

DATA SHARING STATEMENT

The data that support the findings of this study are available from the South African National Health Laboratory Services (NHLS) centralized data warehouse (CDW) and McCord Hospital but restrictions apply to the availability of these data, which were used under license for the current study, and so are not publicly available. Data are however available from the authors upon reasonable request and with permission of the NHLS CDW and McCord Hospital.

For peer review only

For peer review only - http://bmjopen.bmj.com/site/about/guidelines.xhtml

ACKNOWLEDGEMENTS

We gratefully acknowledge the extensive efforts of the clinical and research teams at Sinikithemba for providing strong leadership during a time of challenging transition.

For peer review only

Table 1. Best Match of NHLS Data with McCord Data Solely Using Patient Identifiers

Matching category (general and specific)	Total = 3906
**Confident**	**3278 (83.9%)**
Exact match on surname, first name, DOB*, gender	1823 (46.7%)
Exact match on surname, at least first word of first name, DOB, gender	1433 (36.7%)
Exact match on surname, first name, gender, DOB missing or unusable	8 (0.2%)
Exact match on at least first word of surname and first names, DOB, gender	5 (0.1%)
Exact match on at least first word of surname and first names, gender, DOB missing or unusable	9 (0.2%)
**Likely**	**1 (0.03%)**
Surname and first name are reversed, exact match on gender, DOB missing or unusable	1 (0.03%)
**Likely despite keying errors**	**44 (1.1%)**
Exact match on surname, first name, DOB, gender different	15 (0.4%)
Exact match on surname, first name, gender, DOB discrepant in one part (day, month, or year)	7 (0.2%)
Exact match on surname, at least first word of first name, DOB, gender different	13 (0.3%)
Exact match on surname, at least first word of first name, gender, DOB discrepant in one part (day, month, or year)	9 (0.2%)
**Possible (manually confirmed "yes")**	**150 (3.8%)**
Exact match on at least first word of surname, first word of first name does not match, exact match on DOB (if usable) and gender (if usable)	119 (3.0%)
First word of surname does not match, exact match on at least first word of first name, DOB (if usable) and gender (if usable)	31 (0.8%)
**Other (manually confirmed "yes")**	**16 (0.4%)**
**Possible (manually confirmed "no")**	**3 (0.08%)**
First word of surname does not match, exact match on at least first word of first name, DOB (if usable) and gender (if usable)	3 (0.08%)
**Other (manually confirmed "no")**	**6 (0.2%)**
**No NHLS records**	**408 (10.4%)**

*DOB: date of birth.

For peer review only - http://bmjopen.bmj.com/site/about/guidelines.xhtml

Table 2. NHLS Match for Specific CD4 Test Result and Date in the McCord Data Set

Matching category (general and specific)	Total = 3905
**Confident**	**3270 (83.7%)***
Exact match on CD4 count and test date	2925 (74.9%)
Exact match on CD4 count, month and day of test date reversed	9 (0.2%)
Exact match on CD4 count, test date within 7 days	272 (7.0%)
Exact match on CD4 count, test date discrepant in one part (day, month, or year)	57 (1.5%)
Exact match on CD4 count and registration date	3 (0.08%)
Exact match on CD4 count, registration date within 7 days	2 (0.05%)
Exact match on CD4 count, registration date discrepant in one part (day, month, or year)	2 (0.05%)
**Possible**	**57 (1.5%)**
Different CD4 counts, exact match on test date	57 (1.5%)
**Unlikely**	**36 (0.9%)**
Exact match on CD4 count, different test date	36 (0.9%)
**No match**	**542 (13.9%)**
Different CD4 counts and different test and registration dates	88 (2.3%)
No CD4 value in NHLS	454 (11.6%)

* Percents are of the total McCord records with CD4 results.

For peer review only - http://bmjopen.bmj.com/site/about/guidelines.xhtml

Table 3. NHLS Match for Specific Viral Load Test Result and Date in the McCord Data Set

Matching category (general and specific)	Total = 3609
**Confident**	**3306 (91.6%)***
Exact match on viral load record and test date	2993 (82.9%)
Exact match on viral load record, month and day of test date reversed	9 (0.2%)
Exact match on viral load record, test date within 7 days	254 (7.0%)
Exact match on viral load record, test date discrepant in one part (day, month, or year)	49 (1.4%)
Exact match on viral load record, registration date discrepant in one part (day, month, or year)	1 (0.03%)
**Possible**	**11 (0.3%)**
Different viral load value, exact match on test date	11 (0.3%)
**Unlikely**	**1 (0.03%)**
Exact match viral load value, different test date	1 (0.03%)
**No match**	**291 (8.1%)**
Different viral load values and different test and registration dates	47 (1.3%)
No viral load value in NHLS	244 (6.8%)

* Percents are of the total McCord records with viral load results.

For peer review only

For peer review only - http://bmjopen.bmj.com/site/about/guidelines.xhtml

Table 4. Quality of Patient Identifier Match for Best Test Record Match

Patient match category	Record match category (CD4 or viral load)*				
	Confident	Possible	Unlikely	No match	Total
Confident	3187 (91.9%)	2 (100%)	9 (100%)	13 (3.1%)	3211 (82.2%)
Likely	1 (0.03%)	0	0	0	1 (0.03%)
Likely despite keying errors	63 (1.8%)	0	0	0	63 (1.6%)
Possible: Yes	185 (5.3%)	0	0	4 (0.9%)	189 (4.8%)
Other: Yes	23 (0.7%)	0	0	0	23 (0.6%)
Possible: No	4 (0.1%)	0	0	0	4 (0.1%)
Other: No	6 (0.2%)	0	0	1 (0.2%)	7 (0.2%)
No NHLS Records	0	0	0	408 (95.8%)	408 (10.4%)
Total	3469	2	9	426	3906

*Percentages are column percentages.

For peer review only

REFERENCES

1. How AIDS changed everything - MDG 6: 15 years, 15 lessons of hope from the AIDS response. UNAIDS; 2015.
2. Country ownership for a sustainable AIDS response: From principles to practice. UNAIDS; 2012.
3. Collins C, Beyrer C. Country ownership and the turning point for HIV/AIDS. Lancet Glob Health 2013;1(6):e319-20.
4. Bekker LG, Venter F, Cohen K, Goemare E, Van Cutsem G, Boulle A, et al. Provision of antiretroviral therapy in South Africa: the nuts and bolts. Antivir Ther 2014;19 Suppl 3:105-16.
5. Cohen T, Murray M, Wallengren K, Alvarez GG, Samuel EY, Wilson D. The prevalence and drug sensitivity of tuberculosis among patients dying in hospital in KwaZulu-Natal, South Africa: A postmortem study. PLoS Med 2010;7(6):e1000296.
6. Sherman GG, Lilian RR, Bhardwaj S, Candy S, Barron P. Laboratory information system data demonstrate successful implementation of the prevention of mother-to-child transmission programme in South Africa. S Afr Med J 2014;104(3 Suppl 1):235-8.
7. Leon N, Mathews C, Lewin S, Osler M, Boulle A, Lombard C. A comparison of linkage to HIV care after provider-initiated HIV testing and counselling (PITC) versus voluntary HIV counselling and testing (VCT) for patients with sexually transmitted infections in Cape Town, South Africa. BMC Health Serv Res 2014;14:350.
8. Hsiao NY, Stinson K, Myer L. Linkage of HIV-infected infants from diagnosis to antiretroviral therapy services across the Western Cape, South Africa. PLoS One 2013;8(2):e55308.
9. Dlamini-Mvelase NR, Werner L, Phili R, Cele LP, Mlisana KP. Effects of introducing Xpert MTB/RIF test on multi-drug resistant tuberculosis diagnosis in KwaZulu-Natal South Africa. BMC Infect Dis 2014;14:442.
10. McLaren ZM, Brouwer E, Ederer D, Fischer K, Branson N. Gender patterns of tuberculosis testing and disease in South Africa. Int J Tuberc Lung Dis 2015;19(1):104-10.
11. Sengayi M, Spoerri A, Egger M, Kielkowski D, Crankshaw T, Cloete C, et al. Record

For peer review only - http://bmjopen.bmj.com/site/about/guidelines.xhtml

linkage to correct under-ascertainment of cancers in HIV cohorts: the Sinikithemba HIV clinic linkage project. Int J Cancer 2016.

12. Johnson LF, Dorrington RE, Laubscher R, Hoffmann CJ, Wood R, Fox MP, et al. A comparison of death recording by health centres and civil registration in South Africans receiving antiretroviral treatment. J Int AIDS Soc 2015;18:20628.
13. Joubert J, Bradshaw D, Kabudula C, Rao C, Kahn K, Mee P, et al. Record-linkage comparison of verbal autopsy and routine civil registration death certification in rural north-east South Africa: 2006-09. Int J Epidemiol 2014;43(6):1945-58.
14. Cloete C, Regan S, Giddy J, Govender T, Erlwanger A, Gaynes MR, et al. The linkage outcomes of a large-scale, rapid transfer of HIV-infected patients from hospital-based to community-based clinics in South Africa. Open Forum Infect Dis 2014;1(2):ofu058.
15. Katz IT, Bogart LM, Cloete C, Crankshaw TL, Giddy J, Govender T, et al. Understanding HIV-infected patients' experiences with PEPFAR-associated transitions at a Centre of Excellence in KwaZulu Natal, South Africa: a qualitative study. AIDS Care 2015;27(10):1298-303.
16. Massad E. Fuzzy logic in action: applications in epidemiology and beyond. Springer Verlag2008.
17. Tanaka K. An introduction to fuzzy logic for practical applications. Springer Verlag1997.
18. Lecher S, Ellenberger D, Kim AA, Fonjungo PN, Agolory S, Borget MY, et al. Scale-up of HIV Viral Load Monitoring--Seven Sub-Saharan African Countries. MMWR Morb Mortal Wkly Rep 2015;64(46):1287-90.

For peer review only

For peer review only - http://bmjopen.bmj.com/site/about/guidelines.xhtml

Supplemental Table 1. Sequence of Matching Criteria for Patient Identifiers

**Confident match**
Exact match on surname, first name, DOB*, gender
Exact match on surname, at least first word of first name, DOB, gender
Exact match on surname, first name and:<br>DOB (gender missing or unusable)<br>Gender (DOB missing or unusable)
Exact match on at least first word of surname and first names, DOB, gender
Exact match on at least first word of surname and first names and:<br>DOB (gender missing or unusable)<br>Gender (DOB missing or unusable)
**Likely match**
Surname and first name are reversed and:<br>Exact match on DOB and gender<br>Exact match on DOB (gender missing or unusable)<br>Exact match on gender (DOB missing or unusable)
First word of surname and first word of first name are reversed and:<br>Exact match on DOB and gender<br>Exact match on DOB (gender missing or unusable)<br>Exact match on gender (DOB missing or unusable)
**Likely match despite keying errors**
Exact match on surname, first name, DOB, gender different
Exact match on surname, first name, gender, DOB discrepant in one part (day, month, or year)
Exact match on surname, at least first word of first name, DOB, gender different
Exact match on surname, at least first word of first name, gender, DOB discrepant in one part (day, month, or year)
Exact match on first word of surname, at least first word of first name, DOB, gender different
Exact match on first word of surname, at least first word of first name, gender, DOB discrepant in one part (day, month, or year)
Surname and first name are reversed, exact match on DOB, gender different
Surname and first name are reversed, exact match on gender, DOB discrepant in one part (day, month, or year)
First word of surname and first word of first name are reversed, exact match on DOB, gender different
First word of surname and first word of first name are reversed, exact match on gender, DOB discrepant in one part (day, month, or year)
**Possible match (manual review required)**
Exact match on at least first word of surname, first word of first name does not match , exact match on DOB (if usable) and gender (if usable)
First word of surname does not match, exact match on at least first word of first name, DOB (if usable) and gender (if usable)
**Other match (manual review required)**

*DOB: date of birth.

For peer review only

Supplemental Table 2. Sequence of Matching Criteria for CD4 and Viral Load (VL) Tests

**Confident match**
Exact match on CD4 or VL value* and McCord test date consistent:
Exact match on test date
Month and day of test date reversed
Test date within 7 days
Test date discrepant in one part (day, month, or year)
Exact match on registration date
Month and day of registration date reversed
Registration date within 7 days
Registration date discrepant in one part (day, month, or year)
**Possible match**
Different CD4 or VL value, exact match on test date
Different CD4 or VL value, exact match on registration date
**Unlikely match**
Exact match on CD4 or VL value, different test date
Exact match on CD4 or VL value, different registration date
**No match**
Different CD4 or VL value, different test and registration dates
No CD4 or VL value in NHLS

* VL values are considered matched on the value in any of the following situations:
1. Both McCord and NHLS records had matching viral load values
2. McCord record had a value of <150 copies/ml and NHLS record value was marked "<150"
3. McCord record had a value of <40 copies/ml and NHLS record value was marked "<40"
4. McCord record had a value of <20 copies/ml and NHLS record value was marked "<20"
5. McCord record value was marked "undetectable" and the NHLS record value was marked "<150", "<40", "<20", or "lower than detectable limit"

For peer review only

BMJ Open

Assessing the Completeness and Accuracy of South African National Laboratory CD4 and Viral Load Data: A Cross-sectional Study

Journal:	*BMJ Open*
Manuscript ID	bmjopen-2018-021506.R1
Article Type:	Research
Date Submitted by the Author:	25-May-2018

Complete List of Authors:	Bassett, Ingrid; Massachusetts General Hospital, Division of Infectious Disease; Harvard Medical School<br>Huang, Mingshu; Massachusetts General Hospital, Division of General Internal Medicine; Massachusetts General Hospital, Biostatistics Center<br>Cloete, Christie; McCord Hospital<br>Candy, Sue; National Health Laboratory Service<br>Giddy, Janet; McCord Hospital<br>Frank, Simone; Massachusetts General Hospital, Division of General Internal Medicine; Massachusetts General Hospital, Medical Practice Evaluation Center<br>Parker, Robert; Massachusetts General Hospital, Biostatistics Center; Harvard Medical School

**Primary Subject Heading**:	HIV/AIDS
Secondary Subject Heading:	Public health, Research methods, HIV/AIDS
Keywords:	South Africa, National Health Laboratory Services (NHLS), national laboratory systems, HIV record matching, data crossmatching method, CD4 and viral load

SCHOLARONE™
Manuscripts

Assessing the Completeness and Accuracy of South African National Laboratory CD4 and Viral Load Data: A Cross-sectional Study

Running head: Evaluation of national laboratory data

Ingrid V. Bassett, MD, MPH,[1,2,3,4,5] Mingshu Huang, PhD,[3,4,6] Christie Cloete, MBChB,[7] Sue Candy, BSc,[8] Janet Giddy, MBChB, MFamMed,[7] Simone C. Frank, BA,[2,3] Robert A. Parker, ScD[3,4,5,6]

[1]Division of Infectious Disease, Massachusetts General Hospital, Boston, Massachusetts, United States of America

[2]Division of General Internal Medicine, Massachusetts General Hospital, Boston, Massachusetts, United States of America

[3]Medical Practice Evaluation Center, Department of Medicine, Massachusetts General Hospital, Boston, Massachusetts, United States of America

[4]Harvard University Center for AIDS Research (CFAR), Boston, Massachusetts, United States of America

[5]Harvard Medical School, Boston, Massachusetts, United States of America

[6]Biostatistics Center, Massachusetts General Hospital, Boston, Massachusetts, United States of America

[7]McCord Hospital, Durban, South Africa

[8]Corporate Data Warehouse, Department of Information Technology, National Health Laboratory Services, Johannesburg, South Africa

For peer review only - http://bmjopen.bmj.com/site/about/guidelines.xhtml

Correspondence:

Ingrid V. Bassett, MD, MPH

Massachusetts General Hospital

50 Staniford Street, 9th Floor

Boston, MA 02114

Tel +1 617 726 0637

IVB: ibassett@partners.org

MH: jenniferhuangmsh@gmail.com

CC: christie_cloete@hotmail.com

SC: sue.candy@nhls.ac.za

JG: janet.giddy@gmail.com

SCF: SCFRANK@mgh.harvard.edu

RAP: RPARKER4@PARTNERS.ORG

Word count: 3,013 (word limit 4,000)

These data were presented in part at the 21st International AIDS Conference (AIDS 2016) July 18-22, 2016 in Durban, South Africa.

Keywords: South Africa, National Health Laboratory Services (NHLS), national laboratory systems, HIV record matching, data crossmatching method, CD4 and viral load

For peer review only - http://bmjopen.bmj.com/site/about/guidelines.xhtml

ABSTRACT (word count: 271; word limit: 300)

Objective: To assess the accuracy of the South African National Health Laboratory Services (NHLS) centralized data warehouse (CDW) using a novel data crossmatching method.

Methods: Adults (≥18y) on antiretroviral therapy who visited a hospital-based HIV clinic in Durban from March-June 2012 were included. We matched patient identifiers, CD4 and viral load (VL) records from the HIV clinic's electronic record with the NHLS CDW according to a set of matching criteria for patient identifiers, test values and test dates. We calculated the matching rates for patient identifiers, CD4 and VL records, and an overall matching rate.

Results: NHLS returned records for 3498 (89.6%) of the 3906 individuals requested. Using our computer algorithm, we confidently matched 3278 patients (83.9% of the total request). Considering less than confident matches as well, and then manually reviewing questionable matches using only patient identifiers, only 9 (0.3% of records returned by NHLS) of the suggested matches were judged incorrect.

Conclusions: We developed a data crossmatching method to evaluate national laboratory data and were able to match almost nine of ten patients with data we expected to find in the NHLS CDW. We found few questionable matches, suggesting that manual review of records returned was not essential. As the number of patients initiating ART in South Africa grows, maintaining a comprehensive and accurate national data repository is of critical importance, since it may serve as a valuable tool to evaluate the effectiveness of the country's HIV care system. This study

For peer review only - http://bmjopen.bmj.com/site/about/guidelines.xhtml

helps validate the use of NHLS CDW data in future research on South Africa's HIV care system and may inform analyses in similar settings with national laboratory systems.

For peer review only - http://bmjopen.bmj.com/site/about/guidelines.xhtml

STRENGTHS AND LIMITATIONS OF THIS STUDY

- This is the first analysis to propose a novel method for examining the completeness and accuracy of records related to HIV care from a national data source.
- We developed a comprehensive and self-contained algorithm using commonly available patient identifiers (first name, surname, date of birth, gender) that may inform future analyses focusing on linkage to and retention in HIV care, and this methodology may also apply to data matching analyses in similar settings, as many sub-Saharan African countries have some sort of national laboratory system.
- NHLS requirements for submitting identifiers with laboratory requisitions during the study period were not strict enough to allow uniformly perfect matching; thus, we had to create extensive matching categories to cover the range of match types and quality.
- While we considered our patient identifier, CD4 and VL test record matching criteria detailed and comprehensive, a different team might develop an alternative set of rules and designations, and classify specific results differently.
- We had a large range of patient identifier matching criteria for what we considered an adequate match; while these criteria were discussed at length, they ultimately were subjective decisions.

For peer review only

For peer review only - http://bmjopen.bmj.com/site/about/guidelines.xhtml

INTRODUCTION

South Africa has the largest HIV treatment program in the world, with > 3.1 million people on antiretroviral therapy (ART) [1]. The government has expanded its national program in recent years in a transition to "country ownership" from the previous non-governmental organizations and private clinics [2-5]. As HIV care transitions to the public sector and the number of patients initiating ART grows, maintaining comprehensive and accurate patient data is of critical importance. Reliable and valid national data becomes increasingly useful for evaluating linkage to and retention in HIV care, for monitoring patients longitudinally across clinic sites, and for assessing the quality of care at the national level.

Patients undergoing HIV treatment at public and semi-private health centers in South Africa have routine blood samples sent to a National Health Laboratory Service (NHLS) laboratory for testing; these data are then stored at a central repository in the NHLS Corporate Data Warehouse (CDW). NHLS data have previously been used to evaluate the effectiveness of certain government-funded HIV programs [6-8], identify patterns of the TB epidemic [9, 10], and determine cancer incidence rates among HIV-infected individuals [11]. CD4 count and viral load (VL) records serve as indicators of being in HIV care, as these are monitored regularly while patients are receiving ART. However, NHLS CDW data have not been assessed to determine utility specifically for identifying and tracking patients in HIV care. While previous studies have compared mortality records between South African civil registration and clinics to evaluate the completeness of national mortality data [12, 13], no such comparison has been performed between CD4 and VL records for patients in HIV care.

For peer review only - http://bmjopen.bmj.com/site/about/guidelines.xhtml

We assessed the completeness and accuracy of the NHLS CDW for tracking patients using a cohort of patients who visited McCord Hospital's HIV clinic during a three-month period just prior to clinic closure due to loss of funding. We present here a method developed to match patients based on McCord Hospital patients' identifiers, CD4 records and VL values prior to transfer to data provided to us by the NHLS.

For peer review only

For peer review only - http://bmjopen.bmj.com/site/about/guidelines.xhtml

METHODS

Study Site

McCord Hospital was a semi-private, general hospital in KwaZulu-Natal serving a predominantly urban population from the greater Durban area. The Sinikithemba HIV clinic at McCord, which became a PEPFAR-funded site in 2004, was an integral part of the South African ART scale-up and initiated over 10 000 patients on ART [14]. Sinikithemba served a predominantly African, Zulu-speaking population. The clinic had a monitoring and evaluation team and an electronic medical record. Due to loss of PEPFAR funding, the clinic closed in 2012.

All patients who returned to the clinic for clinical appointments, laboratory tests, or pharmacy refills March 12-June 30, 2012 were referred for transfer to clinics in the Durban area. Data collected at the time of transfer included name, gender, date of birth, most recent pre-transfer CD4 count and VL values and dates. We have previously reported on the Sinikithemba transfer process evaluating linkage to initial transfer clinic visit and patient attitudes about their transfer experience using telephone surveys and clinic visits [14, 15].

Study Population

We studied adults ≥18 years on ART who visited the HIV clinic during the transfer period. Routinely collected programmatic data were used.

National Health Laboratory Service (NHLS)

For peer review only - http://bmjopen.bmj.com/site/about/guidelines.xhtml

The National Health Laboratory Service (NHLS) was established in 2001 and supports national and provincial health departments in South Africa. It is the largest diagnostic pathology service in the country, providing laboratory and related services to over 80% of the population through a national network of laboratories [6]. The NHLS performs all public sector CD4 and VL monitoring and maintains a CDW that serves as a national repository for laboratory data from the public sector. Healthcare workers at public health facilities complete laboratory requisition forms which accompany each sample submitted to the CDW. All data, including patient identifiers, name of facility, date of sample, and tests requested, are sent to the CDW and are captured electronically by the NHLS information system in real time. The CDW has developed an algorithm which utilizes both rules-based and probabilistic matching based on demographic attributes using fuzzy logic [16, 17]. This is applied to all test data at time of entry and results in a master patient index within the CDW.

Data Collection and Processing

We sent a list of all 4257 McCord Hospital transfer patients with corresponding patient identifiers (first name, surname, date of birth, gender) to the NHLS for matching of laboratory records (Supplementary Figures 1A and 1B). We also included an internal study ID to identify each patient so that the NHLS could determine which records they were providing matched our requested records. The NHLS extracted data in October 2014. McCord Hospital data were matched against the entire CD4 and VL datasets for KwaZulu-Natal Province from November 1, 2010 through October 31, 2014. To minimize the data lost when exchanging between systems, the NHLS has checks in place to ensure that the number of records sent by the LIS (Laboratory Information System) interface are processed into the CDW. In the event of system failures, there

For peer review only - http://bmjopen.bmj.com/site/about/guidelines.xhtml

is the ability to re-que data from the LIS. Trend reporting of test volumes over time also assists with data gaps. To assist with the matching process, we also sent last known CD4 and VL values and dates recorded in the electronic medical record at McCord Hospital. We received two datasets (CD4 count and VL) containing potential matches from the NHLS. These datasets had 16 340 and 18 677 records from 3774 patients. We performed three separate matching analyses using patient identifiers (first name, surname, date of birth, gender), CD4 counts and test dates, and VL values and test dates. In each analysis, we assessed the quality of the match within our internal study ID for each patient; thus, we assessed how well the data provided by the NHLS using their probabilistic matching technique represented a true match. From the original 4257 patient list, duplicated study IDs (n = 12) and patients <18 years on June 30, 2012 (n = 337) were removed prior to matching. Two patients who had neither a CD4 count nor VL record from McCord Hospital were also removed. This left a cohort of 3906 patients to match based on patient identifiers. For the CD4 matching analysis, we removed 1 patient who did not have CD4 data in the McCord database, for a cohort of 3905 patients. We removed 297 patients who did not have VL data in the McCord database (missing viral load data may reflect a test not being performed or patients recently initiated on ART who had not yet met guidelines for undergoing a VL test), resulting in a cohort of 3609 patients for the VL record matching analysis.

Matching of Records between NHLS and McCord Datasets

We performed our matching analysis in three stages; first, we cross-checked patient identifiers between the McCord and NHLS datasets to determine the distribution of optimal identifier matching, using all records for a particular individual prior to clinic closure. Next, we assessed the reported CD4 and VL records separately, independent of patient identifiers. Lastly, we

For peer review only - http://bmjopen.bmj.com/site/about/guidelines.xhtml

considered the best test record match from a particular internal study ID number in conjunction with the patient identifier match for that specific record to determine the overall distribution of matching based on both test records and patient identifiers. In this final matching analysis, the patient identifier match was determined for the better match on either CD4 or VL. If the test match quality was the same, we used the better patient match of the two test records.

Matching Using Patient Identifiers

Within each internal study ID for each patient, we used surname, first name, DOB and gender to assess the quality of the match between the NHLS CDW and the McCord data record. Based on a detailed set of matching criteria (Supplementary Table 1), we classified patient study IDs into five general matching categories: *confident, likely, likely despite keying errors, possible*, and *other*. If corresponding patient identifiers fell into the latter two categories, they were reviewed manually; otherwise they were considered an adequate match and not reviewed. The manual review processes consisted of an independent review by two authors (IVB; SCF), with a third “tiebreaker” review by another author (RAP) for any discordant matching designations.

Matching Based on Test Results

We had a cohort of 3905 patients for the CD4 record matching analysis and 3609 patients for the VL matching analysis. If the CD4 count in the McCord record and a corresponding NHLS CDW record were an exact match, we compared the McCord test data to the two dates provided by the NHLS (test date and record date) for consistency (Supplementary Table 2). When the dates were consistent (exact match; month and day reversed; dates differed by less than 7 days; dates differed by one of year, month, or day), we considered the records a *confident* match. If the CD4

For peer review only - http://bmjopen.bmj.com/site/about/guidelines.xhtml

counts from corresponding McCord and NHLS CDW records differed, but there was an exact match on dates, we considered the records a *possible* match. If the dates were not consistent we considered the records an *unlikely* match, even if the CD4 values matched. Records containing both discrepant CD4 values and mismatching dates were considered no match. Following these same criteria, we categorized corresponding NHLS CDW and McCord VL records as *confident*, *possible*, or *unlikely* matches. Because VL is often reported as undetectable, we had to use a somewhat looser criterion for considering the VL result an exact match (Supplementary Table 2).

Matching Based on Patient Identifier, Conditional on Matching based on a Test Result

After matching CD4 and VL values and dates, we assessed the accuracy of the patient identifier information based on the specific record used for the test matching. When there were equally good matches for both the CD4 and VL test, we used the better of the two patient matches for this classification.

Patient and Public Involvement

Neither patients nor the public were involved in developing this project.

For peer review only

For peer review only - http://bmjopen.bmj.com/site/about/guidelines.xhtml

RESULTS

Cohort Characteristics

Of 3906 participants included in the analysis, 41% of the cohort was male and the median age was 39 (interquartile range [IQR] 34 to 46). The majority of patients had CD4 counts above 200/µl at transfer (> 500/µl 29%, 200-500/µl 55%, < 200/µl 15%), and 84% of patients were known to be virologically suppressed.

Best Patient Identifier Matching

Of 3906 patients, 3498 had one or more records returned by the NHLS. There were a median of 6 records (interquartile range: 5-7) per patient combining both CD4 and VL data; the maximum was 37 records for one individual. 3278 (93.7%) of these 3498 patients were considered *confident* matches. The distribution of patient identifier match categories is included in Table 1. Despite considering multiple potential matching criteria, only 45 additional matches (1.2%; *likely* and *possible* matches) were identified using automated procedures. Most of the additional matches (166; 4.7%) were manually confirmed. Only 9 individuals (5.1%) of 175 who required manual review for the best match were not considered a match. Thus, only 0.3% of 3498 with any records were not considered matches. However, an additional 408 (10.4%) of the patients from McCord's HIV clinic did not have records in the NHLS CDW. Thus, overall we were able to match 89.3% of the patients in the McCord record with patients in the NHLS database, and virtually all of the records (99.7%) returned from NHLS were matches to the McCord patients.

Matching Based on CD4 Test Result and Date

For peer review only - http://bmjopen.bmj.com/site/about/guidelines.xhtml

After removing the 1 patient who did not have a CD4 test result in the McCord dataset, there were 3451 patients who had ≥1 CD4 records found in the NHLS CDW. 3270 (94.8%) of these 3451 patients had CD4 records that were considered a *confident* match. 57 (1.7%) records were considered *possible* matches and 36 (1.0%) were considered *unlikely* matches. There were 88 records (2.5%) which did not match on test value and did not match on test date. The distribution of CD4 record matching is shown in Table 2.

Matching Based on Viral Load Test Result and Date

After removing 297 patients who did not have VL results in the McCord dataset, there were 244 (6.8%) patients who did not have any VLs found in the NHLS CDW. Among the returned records for the remaining 3365 patients, there were 3306 (98.2%) VL records that were considered a *confident* match, 11 (0.3%) that were considered *possible* matches and 1 (0.03%) that was considered an *unlikely* match. There were 47 records (1.4%) which did not match on test value and did not match on test date. The distribution of VL record matching is shown in Table 3.

Quality of Patient Identifier Match for Best Test Record Match

After determining the best match for each test for a specific patient study ID, we assessed how well the patient identifiers matched on the specific test record. Among the 3469 patients with a confident match on CD4 or VL, 3187 patients (91.9%) were also considered a *confident* match on the patient identifiers as well, and overall only 10 (0.3%) of these specific test records were not considered matched on the patient identifiers after manual review. For the confidently matched lab tests, the *possible* matches were found to be valid almost all of the time (185/189,

For peer review only - http://bmjopen.bmj.com/site/about/guidelines.xhtml

97.9%) after manual review, but only 23/29 (79.3%) of the patient classified *other* records were valid matches. Most of the additional 272 matches were validated with manual review (208/272, 76.5%). Overall, we manually reviewed 218 records which were confidently matched on a laboratory test, 10 of which were considered not matched (4.6%). The distribution of patient identifier matches by best test matches is shown in Table 4.

For peer review only

DISCUSSION

We assessed the completeness and accuracy of the NHLS CDW by matching patient identifiers and CD4 and VL test results from a McCord Hospital dataset to data returned by NHLS for these individuals. NHLS returned records for 89.6% of the individuals requested. Importantly, we found a very low false matching rate in the NHLS data, as only 0.3% of the patients identified by NHLS were not the patients from our initial request. These mismatches may have occurred due to incorrect recording in our internal database, in the NHLS database, or incorrect data recorded in the lab requisitions. This low false matching rate suggests that our comprehensive matching process is not needed for record reviews for future work. For the few individual patients with mismatching records, there may be implications for missing results when transferring to a new clinic. If there is tight linkage between the NHLS system and public clinic records, these patients may not be correctly found or linked when entering care at a new clinic. Using only personal identifiers, we confidently matched 3278 of 3906 (83.9%) patients. Ignoring identifiers, we confidently matched 83.7% (3270 of 3905) of patients based on CD4 value and test date, and 91.6% (3306 of 3609) of patients with a VL result from McCord Hospital. Of all patients who had a confident match on either a CD4 or VL test, 91.9% (3187 of 3469) of those specific records were also a confident match using patient identifiers.

Comparing patient identifiers between McCord and NHLS datasets, a vast majority of patients were identified as *confident* matches. Confident matches made up 94% of the matched cohort, while all other matching categories combined (*likely*, *likely despite keying errors*, *possible*, and *other*) comprised only 6%, suggesting that the overall quality of matched records was high.

For peer review only - http://bmjopen.bmj.com/site/about/guidelines.xhtml

While it was valuable to examine all potential match types and ranges of match quality, the extensive matching categories may not be necessary as the NHLS records returned were virtually always (99.7%) the patient for whom we requested data. When analyzing CD4 and VL test results separately, there was a slightly higher confident matching rate (98.2%) for VL results than for CD4 records (94.8%) among those with any results returned by NHLS. Patients considered a *confident* match in the CD4 analysis had to have an exact CD4 value match, while patients in the VL analysis had to exhibit a match in VL status if suppressed or exact VL if not suppressed to be considered a *confident* match. Because VL results for most individuals are grouped into a suppressed category, the CD4 analysis may provide a more accurate matching process due to the more precise measure of CD4 value.

There are several limitations to our record matching method. NHLS requirements for submitting identifiers with laboratory requisitions during the study period were not strict enough to allow uniformly perfect matching; thus, we had to create extensive matching categories to cover the range of match types and quality. While we considered our patient identifier, CD4 and VL test record matching criteria detailed and comprehensive, a different team might develop an alternative set of rules and designations, and classify specific results differently. Additionally, we had a large range of patient identifier matching criteria for what we considered an adequate match; while these criteria were discussed at length, they ultimately were subjective decisions. While we were able to categorize a large proportion of records by our matching algorithm, there were additional records that we manually reviewed. Although some manual matches could potentially have been more accurately resolved by consulting an outside source, we sought to keep the record matching algorithm self-contained to increase the likelihood that this method

For peer review only - http://bmjopen.bmj.com/site/about/guidelines.xhtml

could be used by others. Providing laboratory data to NHLS for the matching process might have improved the ability of the NHLS CDW to identify and match our specific patients, so our results might overestimate the ability to match records based solely on patient identifiers. Lastly, while we do not know why 10.6% of individuals requested did not have records returned, we speculate that these individuals may have never had any initial records entered, the data entered may have been so different between NHLS and McCord Hospital that these patients were never identified, or patients may have previously attended a private lab.

Despite the drawbacks of this methodology, this study has several important strengths. This is the first analysis to propose a novel method for examining the completeness and accuracy of records related to HIV care from a national data source. We developed a comprehensive and self-contained algorithm that may inform future analyses focusing on linkage to and retention in HIV care. This methodology may also apply to data matching analyses in similar settings, as many sub-Saharan African countries have some sort of national laboratory system [18]. For this matching analysis, we could only include identifiers that were required on the NHLS laboratory requisition form during the study period (first name, surname, gender, DOB). Adding more required identifiers might increase the utility of national laboratory systems for HIV programs that collect a variety of different identifiers and may also transcend the limitations of using a single official ID, such as South African ID number, for tracking patients across clinics in the public sector. In a previous study where we attempted to collect South African IDs, only a fraction of our participants were able or willing to supply this information and many of the IDs provided were invalid [19]. Lastly, due to the closing of the HIV clinic at McCord Hospital and

For peer review only - http://bmjopen.bmj.com/site/about/guidelines.xhtml

the rapid transfer of a large cohort of patients, we had a considerable number of comprehensive and up-to-date records with which to assess the quality of NHLS CDW data.

For peer review only

For peer review only - http://bmjopen.bmj.com/site/about/guidelines.xhtml

CONCLUSION

As South Africa's HIV treatment program transitions to the public sector and the number of patients initiating ART grows, maintaining a comprehensive and accurate national data repository is of critical importance, as it may serve as a valuable tool to evaluate the effectiveness of the country's HIV care system. Through the method that we created to evaluate national laboratory data, we have demonstrated that the NHLS CDW is both comprehensive and accurate. The NHLS CDW is centralized, broad, and supports a wide coverage of public clinics across the country; it therefore may serve as an appropriate and effective resource for tracking patients within the public HIV care system. Our ability to confirm the NHLS CDW as a reliable data source can help transcend the limitations of collecting and analyzing data within individual clinics, which presents challenges such as differences in record-keeping methods and marked variability in how patients are identified. Health workers, nurses, and clinicians may also be able to use the NHLS to track patients through clinic transfers in the public sector. Additionally, our work suggests that national HIV laboratory systems may benefit from including a more comprehensive set of patient identifiers on laboratory requisition forms to increase the likelihood of containing a complete, accessible list of patients from a wide variety of public HIV programs. This analysis not only validates the use of NHLS CDW data in future studies evaluating South Africa's HIV care system, but may also inform data matching projects in similar settings with national laboratory systems.

For peer review only - http://bmjopen.bmj.com/site/about/guidelines.xhtml

ETHICAL APPROVAL

Participants provided verbal consent for study participation. The study protocol was approved by the McCord Hospital Research Ethics Committee (Durban, South Africa) and the Partners Human Research Committee (2012-P-001122/1, Boston, MA).

For peer review only

For peer review only - http://bmjopen.bmj.com/site/about/guidelines.xhtml

FUNDING

This work was supported by the National Institutes of Health [R01 MH108427 and R01 MH090326-03S1] and the Harvard University Center for AIDS Research [P30 AI060354], which is supported by the following NIH Co-Funding and Participating Institutes and Centers: NIAID, NCI, NICHD, NIDCR, NHLBI, NIDA, NIMH, NIA, NIDDK, NIGMS, NIMHD, FIC, and OAR. The content is solely the responsibility of the authors and does not necessarily represent the official views of the National Institutes of Health.

For peer review only

For peer review only - http://bmjopen.bmj.com/site/about/guidelines.xhtml

COMPETING INTERESTS

The authors have no competing interests to declare.

For peer review only - http://bmjopen.bmj.com/site/about/guidelines.xhtml

For peer review only

AUTHORS' CONTRIBUTIONS

All authors have contributed significantly to this work and have reviewed and approved of this manuscript. IVB, Principal Investigator of this project, led the design and execution of this study as well as all stages of manuscript writing and preparation. MH and RAP led all data analysis efforts. MH initially helped to develop the preliminary novel data crossmatching method, while RAP collaboratively refined the method presented in the manuscript. RAP also contributed substantially to and oversaw all method development. CC and JG both played significant roles in initial data collection and the procurement of records from McCord Hospital. SC also played a significant role in the procurement of CD4 and viral load records from the National Health Laboratory Services, which were used in the data crossmatch. SCF, the Research Assistant, contributed significantly to manuscript writing, editing, and review.

For peer review only - http://bmjopen.bmj.com/site/about/guidelines.xhtml

DATA SHARING STATEMENT

The data that support the findings of this study are available from the South African National Health Laboratory Services (NHLS) centralized data warehouse (CDW) and McCord Hospital but restrictions apply to the availability of these data, which were used under license for the current study, and so are not publicly available. Data are however available from the authors upon reasonable request and with permission of the NHLS CDW and McCord Hospital.

For peer review only

For peer review only - http://bmjopen.bmj.com/site/about/guidelines.xhtml

ACKNOWLEDGEMENTS

We gratefully acknowledge the extensive efforts of the clinical and research teams at Sinikithemba for providing strong leadership during a time of challenging transition.

For peer review only

Table 1. Best Match of NHLS Data with McCord Data Solely Using Patient Identifiers

Matching category (general and specific)	Total = 3906
**Confident**	**3278 (83.9%)**
Exact match on surname, first name, DOB*, gender	1823 (46.7%)
Exact match on surname, at least first word of first name, DOB, gender	1433 (36.7%)
Exact match on surname, first name, gender, DOB missing or unusable	8 (0.2%)
Exact match on at least first word of surname, at least first word of first name, DOB, gender	5 (0.1%)
Exact match on at least first word of surname, at least first word of first name, gender, DOB missing or unusable	9 (0.2%)
**Likely**	**1 (0.03%)**
Surname and first name are reversed, exact match on gender, DOB missing or unusable	1 (0.03%)
**Likely despite keying errors**	**44 (1.1%)**
Exact match on surname, first name, DOB, gender different	15 (0.4%)
Exact match on surname, first name, gender, DOB discrepant in one part (day, month, or year)	7 (0.2%)
Exact match on surname, at least first word of first name, DOB, gender different	13 (0.3%)
Exact match on surname, at least first word of first name, gender, DOB discrepant in one part (day, month, or year)	9 (0.2%)
**Possible (manually confirmed "yes")**	**150 (3.8%)**
Exact match on at least first word of surname, first word of first name does not match, exact match on DOB (if usable) and gender (if usable)	119 (3.0%)
First word of surname does not match, exact match on at least first word of first name, DOB (if usable) and gender (if usable)	31 (0.8%)
**Possible (manually confirmed "no")**	**3 (0.08%)**
First word of surname does not match, exact match on at least first word of first name, DOB (if usable) and gender (if usable)	3 (0.08%)
**Other (manually confirmed "yes")**	**16 (0.4%)**
**Other (manually confirmed "no")**	**6 (0.2%)**
**No NHLS records**	**408 (10.4%)**

*DOB: date of birth.

For peer review only - http://bmjopen.bmj.com/site/about/guidelines.xhtml

Table 2. NHLS Match for Specific CD4 Test Result and Date in the McCord Data Set

**Matching category (general and specific)**	**Total = 3905**
**Confident**	**3270 (83.7%)***
Exact match on CD4 count and test date	2925 (74.9%)
Exact match on CD4 count, month and day of test date reversed	9 (0.2%)
Exact match on CD4 count, test date within 7 days	272 (7.0%)
Exact match on CD4 count, test date discrepant in one part (day, month, or year)	57 (1.5%)
Exact match on CD4 count and registration date	3 (0.08%)
Exact match on CD4 count, registration date within 7 days	2 (0.05%)
Exact match on CD4 count, registration date discrepant in one part (day, month, or year)	2 (0.05%)
**Possible**	**57 (1.5%)**
Different CD4 counts, exact match on test date	57 (1.5%)
**Unlikely**	**36 (0.9%)**
Exact match on CD4 count, different test date	36 (0.9%)
**No match**	**542 (13.9%)**
Different CD4 counts and different test and registration dates	88 (2.3%)
No CD4 value in NHLS	454 (11.6%)

* Percents are of the total McCord records with CD4 results.

For peer review only

For peer review only - http://bmjopen.bmj.com/site/about/guidelines.xhtml

Table 3. NHLS Match for Specific Viral Load Test Result and Date in the McCord Data Set

Matching category (general and specific)	Total = 3609
**Confident**	**3306 (91.6%)***
Exact match on viral load record and test date	2993 (82.9%)
Exact match on viral load record, month and day of test date reversed	9 (0.2%)
Exact match on viral load record, test date within 7 days	254 (7.0%)
Exact match on viral load record, test date discrepant in one part (day, month, or year)	49 (1.4%)
Exact match on viral load record, registration date discrepant in one part (day, month, or year)	1 (0.03%)
**Possible**	**11 (0.3%)**
Different viral load value, exact match on test date	11 (0.3%)
**Unlikely**	**1 (0.03%)**
Exact match viral load value, different test date	1 (0.03%)
**No match**	**291 (8.1%)**
Different viral load values and different test and registration dates	47 (1.3%)
No viral load value in NHLS	244 (6.8%)

* Percents are of the total McCord records with viral load results.

For peer review only

For peer review only - http://bmjopen.bmj.com/site/about/guidelines.xhtml

Table 4. Quality of Patient Identifier Match for Best Test Record Match

Patient match category	Record match category (CD4 or viral load)*				
	Confident	Possible	Unlikely	No match	Total
Confident	3187 (91.9%)	2 (100%)	9 (100%)	13 (3.1%)	3211 (82.2%)
Likely	1 (0.03%)	0	0	0	1 (0.03%)
Likely despite keying errors	63 (1.8%)	0	0	0	63 (1.6%)
Possible: Yes	185 (5.3%)	0	0	4 (0.9%)	189 (4.8%)
Possible: No	4 (0.1%)	0	0	0	4 (0.1%)
Other: Yes	23 (0.7%)	0	0	0	23 (0.6%)
Other: No	6 (0.2%)	0	0	1 (0.2%)	7 (0.2%)
No NHLS Records	0	0	0	408 (95.8%)	408 (10.4%)
Total	3469	2	9	426	3906

*Percentages are column percentages.

For peer review only

For peer review only - http://bmjopen.bmj.com/site/about/guidelines.xhtml

REFERENCES

1. How AIDS changed everything - MDG 6: 15 years, 15 lessons of hope from the AIDS response. UNAIDS; 2015.

2. Country ownership for a sustainable AIDS response: From principles to practice. UNAIDS; 2012.

3. Collins C, Beyrer C. Country ownership and the turning point for HIV/AIDS. Lancet Glob Health 2013 Dec;1(6):e319-20.

4. Bekker LG, Venter F, Cohen K, Goemare E, Van Cutsem G, Boulle A, et al. Provision of antiretroviral therapy in South Africa: the nuts and bolts. Antivir Ther 2014;19 Suppl 3:105-16.

5. Cohen T, Murray M, Wallengren K, Alvarez GG, Samuel EY, Wilson D. The prevalence and drug sensitivity of tuberculosis among patients dying in hospital in KwaZulu-Natal, South Africa: A postmortem study. PLoS Med 2010;7(6):e1000296.

6. Sherman GG, Lilian RR, Bhardwaj S, Candy S, Barron P. Laboratory information system data demonstrate successful implementation of the prevention of mother-to-child transmission programme in South Africa. S Afr Med J 2014 Mar;104(3 Suppl 1):235-8.

7. Leon N, Mathews C, Lewin S, Osler M, Boulle A, Lombard C. A comparison of linkage to HIV care after provider-initiated HIV testing and counselling (PITC) versus voluntary HIV counselling and testing (VCT) for patients with sexually transmitted infections in Cape Town, South Africa. BMC Health Serv Res 2014;14:350.

8. Hsiao NY, Stinson K, Myer L. Linkage of HIV-infected infants from diagnosis to antiretroviral therapy services across the Western Cape, South Africa. PLoS One 2013;8(2):e55308.

For peer review only - http://bmjopen.bmj.com/site/about/guidelines.xhtml

9. Dlamini-Mvelase NR, Werner L, Phili R, Cele LP, Mlisana KP. Effects of introducing Xpert MTB/RIF test on multi-drug resistant tuberculosis diagnosis in KwaZulu-Natal South Africa. BMC Infect Dis 2014;14:442.

10. McLaren ZM, Brouwer E, Ederer D, Fischer K, Branson N. Gender patterns of tuberculosis testing and disease in South Africa. Int J Tuberc Lung Dis 2015 Jan;19(1):104-10.

11. Sengayi M, Spoerri A, Egger M, Kielkowski D, Crankshaw T, Cloete C, et al. Record linkage to correct under-ascertainment of cancers in HIV cohorts: the Sinikithemba HIV clinic linkage project. Int J Cancer 2016 Apr 21.

12. Johnson LF, Dorrington RE, Laubscher R, Hoffmann CJ, Wood R, Fox MP, et al. A comparison of death recording by health centres and civil registration in South Africans receiving antiretroviral treatment. J Int AIDS Soc 2015;18:20628.

13. Joubert J, Bradshaw D, Kabudula C, Rao C, Kahn K, Mee P, et al. Record-linkage comparison of verbal autopsy and routine civil registration death certification in rural north-east South Africa: 2006-09. Int J Epidemiol 2014 Dec;43(6):1945-58.

14. Cloete C, Regan S, Giddy J, Govender T, Erlwanger A, Gaynes MR, et al. The linkage outcomes of a large-scale, rapid transfer of HIV-infected patients from hospital-based to community-based clinics in South Africa. Open Forum Infect Dis 2014 Sep;1(2):ofu058.

15. Katz IT, Bogart LM, Cloete C, Crankshaw TL, Giddy J, Govender T, et al. Understanding HIV-infected patients' experiences with PEPFAR-associated transitions at a Centre of Excellence in KwaZulu Natal, South Africa: a qualitative study. AIDS Care 2015;27(10):1298-303.

16. Massad E. Fuzzy logic in action: applications in epidemiology and beyond. Springer Verlag2008.

For peer review only - http://bmjopen.bmj.com/site/about/guidelines.xhtml

17. Tanaka K. An introduction to fuzzy logic for practical applications. Springer Verlag1997.

18. Lecher S, Ellenberger D, Kim AA, Fonjungo PN, Agolory S, Borget MY, et al. Scale-up of HIV Viral Load Monitoring--Seven Sub-Saharan African Countries. MMWR Morb Mortal Wkly Rep 2015 Nov 27;64(46):1287-90.

19. Bassett IV, Coleman SM, Giddy J, Bogart LM, Chaisson CE, Ross D, et al. Sizanani: A randomized trial of health system navigators to improve linkage to HIV and TB care in South Africa. J Acquir Immune Defic Syndr 2016 Oct 01;73(2):154-60.

For peer review only

Supplementary Table 1. Sequence of Matching Criteria for Patient Identifiers

**Confident match**
Exact match on surname, first name, DOB*, gender
Exact match on surname, at least first word of first name, DOB, gender
Exact match on surname, first name and:<br>DOB (gender missing or unusable)<br>Gender (DOB missing or unusable)
Exact match on at least first word of surname, at least first word of first name, DOB, gender
Exact match on at least first word of surname, at least first word of first name and:<br>DOB (gender missing or unusable)<br>Gender (DOB missing or unusable)
**Likely match**
Surname and first name are reversed and:<br>Exact match on DOB and gender<br>Exact match on DOB (gender missing or unusable)<br>Exact match on gender (DOB missing or unusable)
First word of surname and first word of first name are reversed and:<br>Exact match on DOB and gender<br>Exact match on DOB (gender missing or unusable)<br>Exact match on gender (DOB missing or unusable)
**Likely match despite keying errors**
Exact match on surname, first name, DOB, gender different
Exact match on surname, first name, gender, DOB discrepant in one part (day, month, or year)
Exact match on surname, at least first word of first name, DOB, gender different
Exact match on surname, at least first word of first name, gender, DOB discrepant in one part (day, month, or year)
Exact match on first word of surname, at least first word of first name, DOB, gender different
Exact match on first word of surname, at least first word of first name, gender, DOB discrepant in one part (day, month, or year)
Surname and first name are reversed, exact match on DOB, gender different
Surname and first name are reversed, exact match on gender, DOB discrepant in one part (day, month, or year)
First word of surname and first word of first name are reversed, exact match on DOB, gender different
First word of surname and first word of first name are reversed, exact match on gender, DOB discrepant in one part (day, month, or year)
**Possible match (manual review required)**
Exact match on at least first word of surname, first word of first name does not match , exact match on DOB (if usable) and gender (if usable)
First word of surname does not match, exact match on at least first word of first name, DOB (if usable) and gender (if usable)
**Other match (manual review required)**

*DOB: date of birth.

For peer review only

Supplementary Table 2. Sequence of Matching Criteria for CD4 and Viral Load (VL) Tests

**Confident match**
Exact match on CD4 or VL value* and McCord test date consistent:
Exact match on test date
Month and day of test date reversed
Test date within 7 days
Test date discrepant in one part (day, month, or year)
Exact match on registration date
Month and day of registration date reversed
Registration date within 7 days
Registration date discrepant in one part (day, month, or year)
**Possible match**
Different CD4 or VL value, exact match on test date
Different CD4 or VL value, exact match on registration date
**Unlikely match**
Exact match on CD4 or VL value, different test date
Exact match on CD4 or VL value, different registration date
**No match**
Different CD4 or VL value, different test and registration dates
No CD4 or VL value in NHLS

* VL values are considered matched on the value in any of the following situations:
1. Both McCord and NHLS records had matching viral load values
2. McCord record had a value of <150 copies/ml and NHLS record value was marked “<150”
3. McCord record had a value of <40 copies/ml and NHLS record value was marked “<40”
4. McCord record had a value of <20 copies/ml and NHLS record value was marked “<20”
5. McCord record value was marked “undetectable” and the NHLS record value was marked “<150”, “<40”, “<20”, or “lower than detectable limit”

For peer review only

SUPPLENTARY FIGURE LEGEND

Supplementary Figure 1A. Process of Determining Cohorts for Crossmatching Analysis

We started with a patient list of 4257 McCord Hospital study IDs. Prior to matching with NHLS data, we removed duplicated study IDs (n=12), patients <18 years old on June 30, 2012 (n=337), and patients who had neither a CD4 count nor VL record from McCord Hospital (n=2), leaving a cohort of 3906 patients for patient identifier matching ("Filter 1"). For the CD4 matching analysis, we then removed a patient who did not have a CD4 count record from McCord Hospital (n=1), leaving a cohort of 3905 patients for CD4 matching ("Filter 2"). For the VL matching analysis, we removed 297 patients who did not have a VL record from McCord Hospital, leaving a cohort of 3609 for VL matching ("Filter 3").

Abbreviations: **NHLS:** National Health Laboratory Services; **VL:** viral load.

Supplementary Figure 1B. Process of Receiving NHLS Data for Crossmatching Analysis

We sent 4257 McCord Hospital study IDs to the NHLS. Study IDs were sent with associated patient identifiers (first name, surname, gender, date of birth) and last recorded CD4/VL from McCord Hospital. The NHLS then returned 3774 study IDs; the returned dataset contained 16 340 CD4 records and 18 677 VL records from 3774 patients. We then compared these 3774 study IDs to each of our filtered cohorts (Supplemental Figure 1A). Of our 3906 cohort for patient identifier matching, 3498 had one or more records returned by NHLS. Of our 3905 cohort for CD4 matching, 3451 had one or more CD4 records in NHLS. Of our 3609 cohort for VL matching, 3365 had one or more VL records in NHLS.

Abbreviations: **NHLS:** National Health Laboratory Services; **VL:** viral load.

For peer review only

Supplementary Figure 1A.

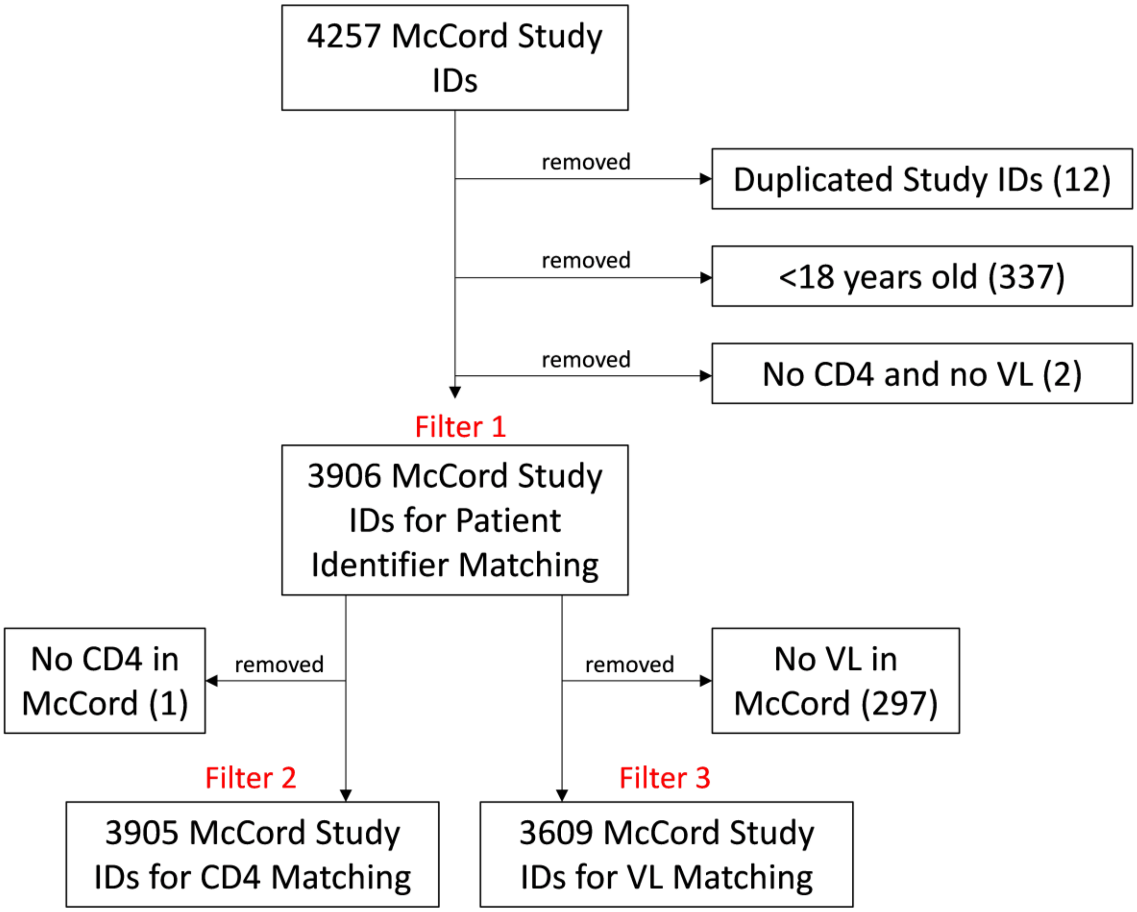

Supplementary Figure 1B.

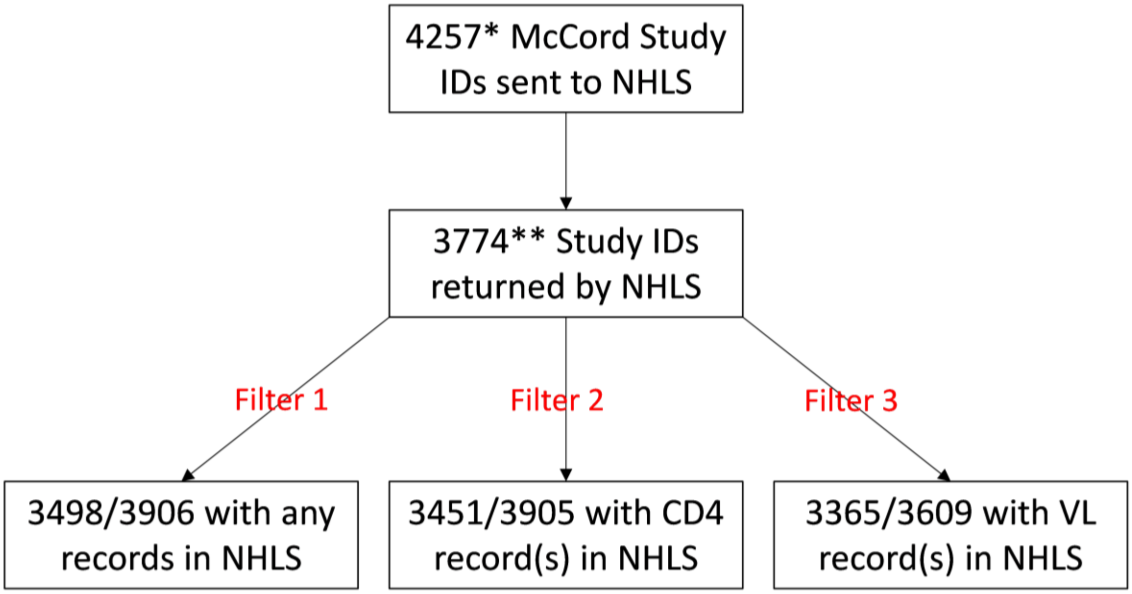

*Study IDs sent with associated patient identifiers and last recorded CD4/VL from McCord Hospital.
**Dataset contained 16 340 CD4 records and 18 677 VL records from 3774 patients.

STROBE Statement—Checklist of items that should be included in reports of ***cross-sectional studies***

	Item No	Recommendation	Page/Line # in manuscript
**Title and abstract**	1	(*a*) Indicate the study's design with a commonly used term in the title or the abstract	1/1
		(*b*) Provide in the abstract an informative and balanced summary of what was done and what was found	3-4/47-71
**Introduction**			
Background/rationale	2	Explain the scientific background and rationale for the investigation being reported	6/118-138
Objectives	3	State specific objectives, including any prespecified hypotheses	7/140-144
**Methods**			
Study design	4	Present key elements of study design early in the paper	
Setting	5	Describe the setting, locations, and relevant dates, including periods of recruitment, exposure, follow-up, and data collection	8/164-178
Participants	6	(*a*) Give the eligibility criteria, and the sources and methods of selection of participants	8/180-182
Variables	7	Clearly define all outcomes, exposures, predictors, potential confounders, and effect modifiers. Give diagnostic criteria, if applicable	9-11/198-265
Data sources/ measurement	8*	For each variable of interest, give sources of data and details of methods of assessment (measurement). Describe comparability of assessment methods if there is more than one group	9-10/198-222
Bias	9	Describe any efforts to address potential sources of bias	17-18/380-398
Study size	10	Explain how the study size was arrived at	9-10/198-222
Quantitative variables	11	Explain how quantitative variables were handled in the analyses. If applicable, describe which groupings were chosen and why	9-11/198-265
Statistical methods	12	(*a*) Describe all statistical methods, including those used to control for confounding	10-12/224-265
		(*b*) Describe any methods used to examine subgroups and interactions	N/A
		(*c*) Explain how missing data were addressed	10/214-222
		(*d*) If applicable, describe analytical methods taking account of sampling strategy	N/A
		(*e*) Describe any sensitivity analyses	N/A
**Results**			
Participants	13*	(a) Report numbers of individuals at each stage of study—eg numbers potentially eligible, examined for eligibility, confirmed eligible, included in the study, completing follow-up, and analysed	13-15/285-327
		(b) Give reasons for non-participation at each stage	N/A
		(c) Consider use of a flow diagram	Supplementary Figure
Descriptive data	14*	(a) Give characteristics of study participants (eg demographic, clinical, social) and information on exposures and potential confounders	13-15/278-283
		(b) Indicate number of participants with missing data for each variable of interest	Supplementary Figure

For peer review only

For peer review only - http://bmjopen.bmj.com/site/about/guidelines.xhtml

Outcome data	15*	Report numbers of outcome events or summary measures	N/A
Main results	16	(*a*) Give unadjusted estimates and, if applicable, confounder-adjusted estimates and their precision (eg, 95% confidence interval). Make clear which confounders were adjusted for and why they were included	N/A
		(*b*) Report category boundaries when continuous variables were categorized	N/A
		(*c*) If relevant, consider translating estimates of relative risk into absolute risk for a meaningful time period	N/A
Other analyses	17	Report other analyses done—eg analyses of subgroups and interactions, and sensitivity analyses	13-15/278-283
**Discussion**			
Key results	18	Summarise key results with reference to study objectives	16/348-363
Limitations	19	Discuss limitations of the study, taking into account sources of potential bias or imprecision. Discuss both direction and magnitude of any potential bias	17-18/380-398
Interpretation	20	Give a cautious overall interpretation of results considering objectives, limitations, multiplicity of analyses, results from similar studies, and other relevant evidence	20/439-454
Generalisability	21	Discuss the generalisability (external validity) of the study results	20/439-454
**Other information**			
Funding	22	Give the source of funding and the role of the funders for the present study and, if applicable, for the original study on which the present article is based	22/485-490

*Give information separately for exposed and unexposed groups.

Note: An Explanation and Elaboration article discusses each checklist item and gives methodological background and published examples of transparent reporting. The STROBE checklist is best used in conjunction with this article (freely available on the Web sites of PLoS Medicine at http://www.plosmedicine.org/, Annals of Internal Medicine at http://www.annals.org/, and Epidemiology at http://www.epidem.com/). Information on the STROBE Initiative is available at www.strobe-statement.org.

For peer review only
